# Bridging the gap: fostering interactive stimming between non-speaking autistic children and their parents

**DOI:** 10.3389/fnint.2024.1374882

**Published:** 2024-05-22

**Authors:** Rachel S. Y. Chen

**Affiliations:** College of Humanities, Arts and Social Sciences, Linguistics and Multilingual Studies, Nanyang Technological University, Singapore, Singapore

**Keywords:** autism, neurodiversity, embodiment, social interaction, repetitive behavior and stimming, music, Augmentative and Alternative Communication (AAC), Design-Based Research

## Abstract

Non-speaking autistic individuals grapple with the profound impact of the ‘double empathy problem’ in their daily interactions with speaking others. This study rethinks the communication challenges faced by non-speaking autistic individuals, challenging traditional approaches that predominantly focus on speech and complex communication devices (AAC). By spotlighting the natural phenomenon of “interactive stimming,” a powerful mode of communication among autistic individuals, this study advocates for a shift from a conventional emphasis on speech towards the foundational role of the body in autistic communication. Central to this exploration is the introduction of the Magical Musical Mat (MMM), an innovative interactive environment mapping interpersonal touch to musical sounds. Through a robust mixed-methods approach integrating video-based fieldwork and design-based research, this paper engages three non-speaking autistic children and their mothers in a 5-day empirical intervention. Results reveal significant transformations in parent–child interactions as both parents and children are acquainted with touch in a new environment. Children assert their autonomy, exploring at their own pace, and discovering sensory features of the environment. Notably, the introduction of sound prompts heightened awareness of the stims, leading to diversified and expressive stim movements. Foregrounding interpersonal touch eventually guides parents into their children’s sensory activities where parents attune to the stims of their children by joining in and facilitating their expressiveness, co-creating extended, evolving patterns of repetitive cycles. The collaborative stim cycles can be likened to free improvisation, where dynamical coherence between individuals occurs through a blend of stability and active flexibility. By shifting the focus from speech to co-created sensory experiences, this paper sheds light on the value of transformative multisensory environments, envisioning a world where varied modes of communication are valued and embraced.

## 1 Introduction

Originally characterized as a disorder of “affective disturbances” by Kanner (1943), Autism from the medical perspective is a neurocognitive disability clinically defined by deficits in social interaction and communication, and the manifestation of restrictive repetitive behaviors, routines, and interests (DSM-5, American Psychiatric Association, 2013). With the growth of the neurodiversity movement (Kapp, 2020; Botha et al., 2024) and the shift away from deficit-oriented perspectives around disability, Autism has been reconceptualized as a neurological variation (Walker, 2014), revealing the infinite potential and diversity of the human mind (Solomon, 2010), and opening doors to a holistic understanding of human cognition, communication, and perception (De Jaegher, 2013). This paradigm shift has prompted a reconsideration of services, learning environments, and instruction away from primarily behavioral interventions, and instead towards embracing playful, exploratory learning with the aim of self-discovery (Torres et al., 2023).

Many testimonies of autistic^1^ people have contradicted accounts of the autistic communicative deficit, expressing the need for a sense of belonging (Mitchell et al., 2021), as well as the desire for friendship, even in childhood (Conn, 2015). Over the last decade, there has been a notable shift in scientific discourse concerning Autism. Rather than solely attributing the social difficulties of autistic individuals to a “lack of social intent,” researchers have begun to conceptualize these challenges as stemming from disparities in dispositional outlooks during social interaction. The interactions between autistic and non-autistic individuals have been forefronted as the locus of the ‘double empathy problem’: “a disjuncture in reciprocity between two differently disposed social actors” (Milton, 2012, p. 884). This paradigm shift acknowledges the complexities inherent in social interactions, particularly between individuals with varying neurotypes, such as autistic and non-autistic individuals, underscoring the significance of the ‘local interaction order’ as autistic individuals engage in social encounters (Maynard and Turowetz, 2022). The consequences of the double empathy problem are even more pronounced for a significant portion of the autistic population that has limited speech ability due to other co-occurring disabilities. Born into a speech-centric, hearing-privileged world (Savarese, 2021), the production of oral-acoustic speech and the ability to hear the speech of others are ubiquitous prerequisites for social participation, leading to significant ramifications for the communicative practices of non-speaking autistic individuals.

However, every living being is in constant attunement with the world (Merleau-Ponty, 1962), invoking the social and material environment in various ways (Goodwin, 1995, 2004, 2018), interweaving multiple modalities to augment their communication (Savarese, 2021) from the earliest stages of life. Every socially meaningful action—even posture we hold—is responsive to interactional circumstances, the relational history of parties, and the body’s ongoing adaptations to its organic needs (Streeck, 2018). Some of the first and most foundational interactions children experience with family members occur not through speech, but through an embodied choreography of hugs, shepherding, and various touch-based practices as family life is navigated (Goodwin and Cekaite, 2018). Autistic individuals experience a deep sense of permeability with external systems of organization, where cultural materials are sought to “create a sense of coherence, order, safety, and joy” (Fein, 2018; p. 129). Yet, conventions of social and sensory orientations (Broderick, 2008) inadvertently govern the Autistic body as it interacts with the material and social world. When unconventional social actions are produced by non-speaking autistic individuals, they often bring chagrin to non-autistic interactants (Maynard and Turowetz, 2022), especially when these actions do not involve speech (Jaswal and Akhtar, 2019; Jaswal et al., 2020; Chen, 2022a).

Some of the challenges non-speaking autistic individuals encounter in their social interactions with non-autistic individuals stem from their distinct sensorimotor differences, and a pervasive emphasis on speech in their daily interactions. Trevarthen and Delafield-Butt (2013) argue that the socio-emotional differences associated with autistic children and adults are secondary consequences to sensory processing (see also Aron and Aron, 1997; Aron et al., 2012) and affective integration issues. As a result of these sensorimotor differences, the behaviors of autistic individuals have pervasively been spotlighted as displaying a lack of social interest. When participating in social interaction, Autistic interactants may not produce non-verbal and verbal markers of joint attention—shared mutual gaze (Akhtar and Gernsbacher, 2008; Dindar et al., 2017; Korkiakangas, 2018; Akhtar and Jaswal, 2020), pointing (Baron-Cohen, 1989; Korkiakangas, 2018), and discourse markers of attentiveness such as ‘mmhm’—that may be expected by neurotypical interactants, but are less important to autistic interactants (Rifai et al., 2022).

In therapeutic and familial contexts, non-speaking autistic individuals additionally face the expectation that their social actions are participatory only if produced verbally. In the hierarchy of sensory learning, children are expected to transition from sensory and embodied forms of meaning-making, and instead adopt codified signs and symbols of cultural knowledge from the realm of adulthood (Nolan and McBride, 2015). Parents have been found to halt the progressivity of their interaction with their autistic children in favor of them producing the correct utterances, even if the request has been made gesturally (Chen, 2022a). Augmentative and Alternative Communication (AAC) is a staple therapeutic solution for facilitating communication among non-speaking individuals (Zangari et al., 1994). Although useful for many, traditional AAC devices, often speech-generating tools with complex grid-and-symbol interfaces, have proven especially restrictive for autistic individuals, imposing high cognitive and motor demands (Light et al., 2019). Their exclusive focus on generating linguistic forms over interactional function (Yu and Chen, 2024) often limits communication to basic wants and needs (Mirenda, 2008). Furthermore, these devices tend to overlook the body’s rich communicative potential and compel individuals to conform to the preferred communicative medium of their interlocutors. This study sheds light on the often overlooked communicative practices of non-speaking autistic individuals. By challenging the conventional emphasis on speech, this study poses the fundamental question: How can we rethink autistic interactional practices if speech is not the focal point?

Central to this exploration is the phenomenon of “interactive stimming.” Stimming, the repetitive production of sensorimotor movement, is an “intrinsically motivating sensory event” that is a core facet of the Autistic experience (Nolan and McBride, 2015; p. 1075; Torres and Whyatt, 2017). This myriad of movements have a long-held status as solitary behavior, and even as distracting to social interaction (Lewis and Bodfish, 1998; Leekam et al., 2011). Whereas these behaviors are often interpreted as indicating a lack of social interest, they may in fact constitute adaptive responses to the unique circumstances of being autistic (Jaswal and Akhtar, 2019). Stimming has been reported by Autistic individuals to bring both calmness and joy, having utility in both self-regulation and expression (Higashida, 2013; Conn, 2015; Coo, 2018; Kapp et al., 2019). Past empirical work has additionally demonstrated its attunement to larger sequences of interaction (Dickerson et al., 2007; Chen, 2016), its emergence in musical contexts such as the Exploratory World Music Playground (E-WoMP) (Bakan, 2014), and its presence as “interactive stimming” in Autistic culture (Sinclair, 2010; Conn, 2015) as a “natural” and “powerful” means of communicating (Bascom, 2012, p. 25).

Interactive stimming presents an opportunity to diverge from normative sensory ideals, enabling a rethinking of the autistic sensory experience not as a sensory integration disorder, but instead as an integral aspect of autistic embodiment (Nolan and McBride, 2015). In this study, I contend that interactive stimming is fundamental to the social worlds of non-speaking autistic individuals. Through a robust mixed-methods approach, integrating video-based fieldwork and design-based research cycles, the study engages with three non-speaking autistic children with different sensory profiles and their mothers in a five-day empirical intervention. I ask three questions: (1) What tools and environments can better surface the naturally-occurring modes of communication of non-speaking autistic individuals? (2) How can non-autistic interactants join in co-constructing rich, embodied interaction with autistic individuals? (3) What are the interactional structures present in these communicative practices?

## 2 Materials and methods

### 2.1 Participants

The study recruited three children diagnosed with Autism (ages 5–18 years) with minimal to no spoken language production, and their respective parents to participate. The children were recruited from parent support groups and schools, and recruitment was open to the diverse ethnic, cultural, and socioeconomic demographics of local communities in Singapore. With Singaporean-Chinese being the majority race in Singapore, most participants who eventually responded were Chinese, although some Indian and Malay families also responded. All participants came from diverse socioeconomic backgrounds. For this study, the three families involved were Chinese, with one family being both Chinese and Indian. These participants came from mid to high income families, although other recruited participants were involved in a second intervention study that took place later on. Eligibility was determined based on the following conditions: (1) A confirmed clinical diagnosis of Autism, (2) informed consent from both parent/guardian and child, and (3) minimal to non-existent spoken language production.

The parent–child dyads in the study were Matt (14-years-old) and his mother, Nathan (12-years-old) and his mother, and Chloe (5-years-old) and her mother. Matt and Chloe are Singaporean-Chinese, and Nathan is Singaporean-Indian-Chinese. All participants use a combination of Singaporean English and Singapore Colloquial English (Leimgruber, 2013) as the primary spoken languages at home, and some of Chloe’s family members use Mandarin Chinese, although they speak to her mostly in English. All the children’s mothers were the ones who first contacted me, and the family members who themselves participated in the study. All three children were in Special Education at the time. Nathan and Chloe were additionally receiving Speech Therapy, and Matt was receiving a movement-based therapy based on Feldenkrais.

At home, Matt communicated most frequently with his mother on a low-tech AAC device—an alphabet board—where he would spell out words and sentences to her. Nathan did not have any assistive communication device that allowed for the production of words. Chloe used a high-tech AAC device that housed a minspeak system. According to her mother, and throughout the study, Chloe used the device to request for food and activities.

When I came into contact with the parents, I collaborated with them to find a time in their schedule that would be ideal. According to prior work on video-based fieldwork with non-speaking autistic children, unplanned, ‘free’ time would occur between larger activity junctures, for example, between mealtime and having to leave the house for therapy (Chen, 2022a). The team visited the families during these times over 5 days in a span of 1–2 weeks.

### 2.2 Creation of novel artifacts

The Magical Musical Mat (MMM) is an interactive environment that maps interpersonal touch to dynamically changing music and sound (Chen et al., 2020, 2024). When two people sit, lie, or stand on floormats and establish skin contact with one another, they close and thus activate an electronic circuit. Capacitive sensors—a conductive rubber called “velostat”—in the mat detect their co-produced touch actions, triggering a variety of sounds. Different types of touch, such as holding hands, high-fives, or gentle taps, dynamically and spontaneously change auditory qualities, resulting in a rich diversity of sound-touch expression. Details of the interactive system’s design and development are discussed in Chen et al., (2024). Influenced by Tangible User Interfaces (Ryokai et al., 2004; Ishii, 2008), the MMM draws inspiration from copresence through touch and intercorporeality (Merleau-Ponty, 1962), as well as research on family touch practices (Goodwin, 2000; Goodwin and Cekaite, 2018; Katila, 2018).

**Figure 1:**
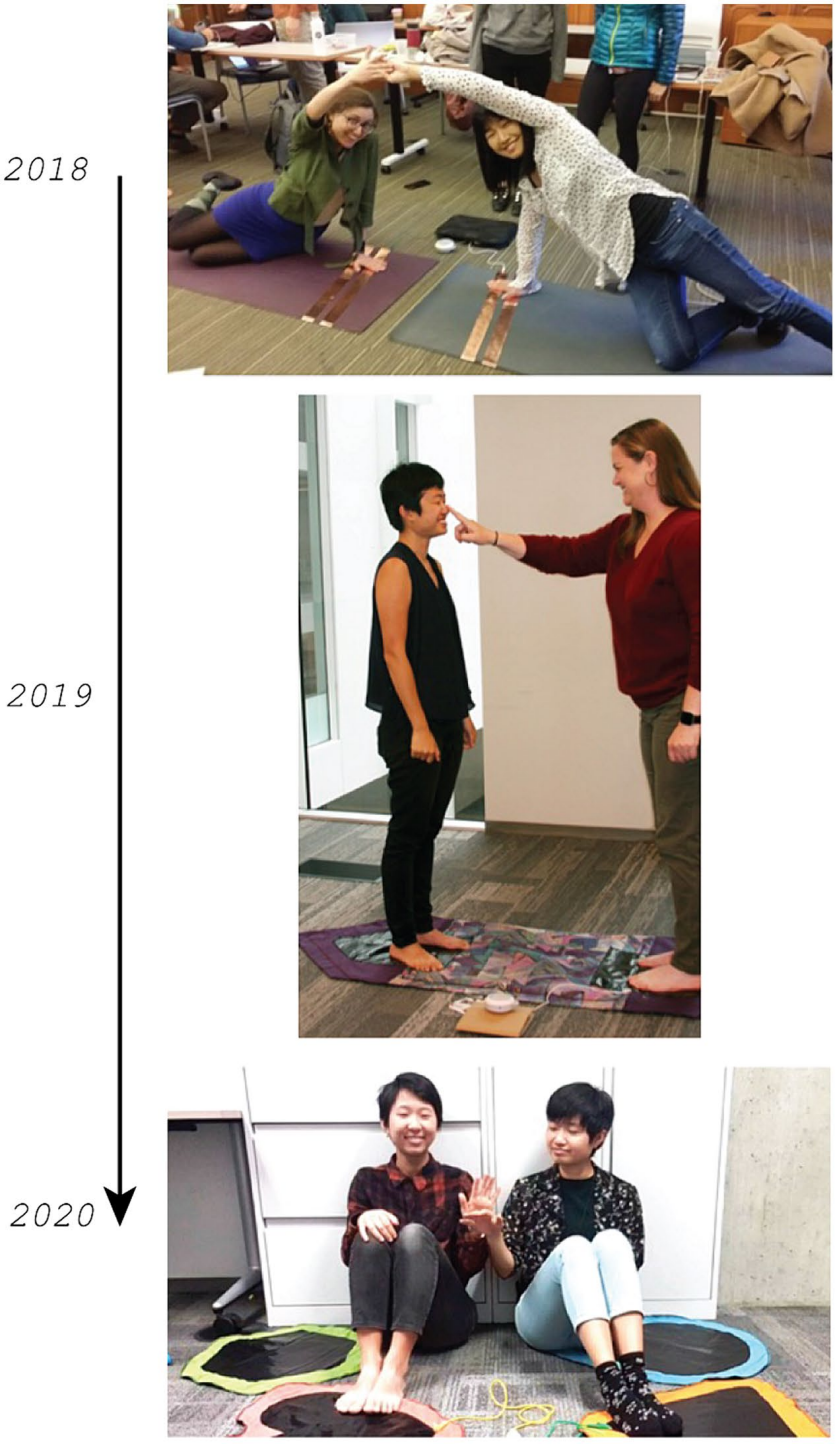
Timeline of Magical Musical Mat form factor. Image one includes Noura Houwell and Kimiko Ryokai touching copper tapes on yoga mats with one hand, then reaching out and touching each other. Image two was first published in the BSE newsletter entitled “Engaging Autistic Students Through Music” by Ellen Lee, featuring the author (Rachel) and Tara Kaiser standing on a long mat with Tara touching Rachel’s nose. Image three involves Rachel and Arianna Ninh sitting on various colored mats and touching hands.

The initial iteration of MMM was not designed with autistic children in mind, but rather for novel and joyful interaction between people. Its adoption emerged from video-based fieldwork with autistic individuals which highlighted their naturally occurring interpersonal touch interactions with peers or siblings (Chen, 2021; Chen and Cekaite, 2021). In Chen (2021), a pair of siblings involving a non-speaking autistic child were found to participate in cycles of rich touch-based interactions with one another, touching hands, rubbing noses and cheeks with one another, and producing complex interactions that were playful. Chen and Cekaite (2021) details a non-speaking autistic child’s interactions with his older friend. They transform the child’s favorite object—a comb—into an object of play as the child reaches for it, requests to be carried by his friend, and tries to grab it while being carried. These interactions tended to occur not between autistic children and their parents, but rather between autistic children and their siblings and friends. The MMM was adopted as an opportunity to enable playful touch-based interactions between autistic individuals and others, such as their parents, teachers, or peers. Autistic individuals’ unique affinity with music (Janzen and Thaut, 2018; Chen, 2022b) informed the development of MMM as a communicative therapeutic tool. Initial iterations involved collaboration with an autism clinic (Chen et al., 2020). Figure 1 shows a timeline of the Magical Musical Mat form factor.

### 2.3 Study procedure

This study integrates two approaches, namely video-based fieldwork, and design-based research. Video ethnography centered around studying situated human interaction is an approach that involves setting up cameras or entering a research site with a “roving camera” (Heath et al., 2010, p. 38). Video recordings provide evidence and accurate grounds for observable phenomena (Garfinkel, 1967; Sacks, 1992), and additionally allow the research team to evaluate their own interactional practices with participants. In this study, two stationary cameras were set up at corners of the participants’ living rooms, and a roving camera was held by at least one research team member. The video data was supplemented with an ethnographic interview with each mother on the first day of the study.

Design-based research (DBR) integrates cycles of design, implementation, and evaluation to generate and generalize theory in educational research (Collins, 1992; Bakker, 2018). Focused on “ontological innovation,” DBR develops explanatory constructs and causal accounts, informing the creation of design solutions. This practice yields contributions in theoretical constructs, validated educational artifacts, and heuristic design frameworks (Cobb et al., 2003; Abrahamson, 2015).

The study underwent three iterative cycles with the three autistic children and their families. The first cycle involved Matt, the second cycle involved Nathan, and the last cycle involved Chloe. Each cycle spanned 10 days, over five separate sessions. Changes between each cycle include improvements to the form factor of the MMM mats, tweaks to the sounds of the MMM, and refinements to our own interactional practices with participants.

The study procedure aligns with the E-WoMP, which advocates for an ethnomethodology of autism that privileges ability over disability, and differences and diversity over unusualness (Bakan et al., 2008). The ethnomusical orientation prioritizes music-play that is spontaneous and unstructured, free of specific goals and demands whether musical or social.

**Figure 2:**
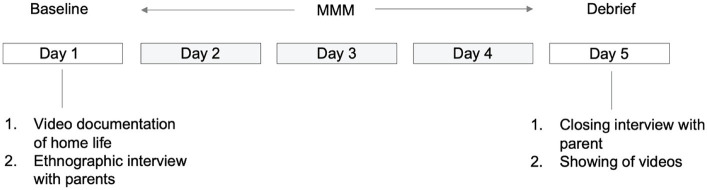
Structure of intervention cycle with each family.

Each iterative cycle followed a three-part structure (Figure 2). In the baseline phase, 1-2 h of the autistic child’s home life during ‘free’, unplanned time was video-recorded by a research assistant and I. During this time, the child’s stimming behavior, interactions with family members, and interactions with material objects and their environment were observed and documented by the research team. A brief ethnographic interview was carried out with the parent during this phase (Appendix 1).

In the intervention phase, three sessions lasting 1–2 h each were conducted with each family. Before each session, video cameras were set up, and the Magical Musical Mat (MMM) was prepared, customized to each child. After informing participants that the mats were ready, they were free to interact while being video-recorded, with minimal guidance. Between sessions, the research team reflected on the experience, making improvements in our interactions, the MMM’s design, and mat placement in the living room. Much like the ethnomusical work with autistic individuals (Bakan et al., 2008), the intervention sessions provided play-sessions with participants, orienting to following the child’s lead, having them direct the course and flow of play. The final session included a close-out interview with parents, showcasing video clips and allowing open discussion for feedback and improvements. Children were encouraged to interact freely during this concluding conversation.

### 2.4 Ongoing adaptations to the intervention

The MMM was continuously enhanced for each family in its form factor, sound output, and also where we would place the mats in the children’s homes. Noteworthy improvements to the MMM system included the addition of longer mats for children to lie down on, adjustments to the sound palette to each child’s musical preferences, and modifications to mat placement in the living rooms (see Table 1). Because Matt enjoyed lying down on the mats, we included longer mats for Nathan and Chloe, our second and third participants, so that they had a variety of mat sizes to choose from and could subsume a diversity of postures on the mats.

**Table 1:** Adaptations to the Magical Musical Mat.

	Matt	Nathan	Chloe
Day 2	Sound palette: Marimba Mat placement: *On the floor, under the living room fan.*	Sound palette: Marimba Mat placement: *On the floor, under the living room fan.*	Sound palette: Marimba Mat placement: *On the floor, under the living room fan.*
Day 3	Sound palette: Marimba Drums Mat placement: *On the floor in front of the sofa.*	Sound palette: Sustained-chords Drums Marimba Mat placement: *On the floor.* *On the swing.*	Sound palette: Marimba Ode to Joy Drums Mat placement: *On the floor, under the living room fan.*
Day 4	Sound palette: Marimba Mat placement: *On the floor in front of the sofa.*	Sound palette: Sustained-chords Marimba Mat placement: *On the floor.* *On the sofa.* *On the floor.*	Sound palette; Ode to Joy Star Wars Ode to Joy Mat placement: *On the floor, under the living room fan.*

Being a team of speaking individuals working with non-speaking individuals, interactions with participants on site, as well as the introduction of new materials into the child’s home require care, sensitivity and consistent reflection. Reflexive analysis of video recordings facilitated ongoing improvements in researcher interactions throughout the study. Throughout each family cycle, our interactional practices underwent changes, considering ethical implications and participant roles (Chen, 2021; Edmonds, 2021). Following the touch sensitivities detailed in Fitzgerald (2013), the research team considered each child’s touch preferences and closely monitored the dyads’ unfolding interactions. Significant changes include instructions around how the mat was introduced, particularly to address heavy use of control touch by parents (Cekaite, 2015). Adjustments in verbal instructions aimed to encourage diverse touch-based interactions, emphasizing an open and inviting space.

### 2.5 Data analysis

First, data analysis focused on Day 1 of each cycle, involving video recordings of children’s interactions, stimming practices, and family dynamics, complemented by ethnographic interviews with parents. These analyses were attempts to provide a holistic understanding of the children’s lived experiences before the intervention occurred (see also, Bakan et al., 2008). Second, the study identified the initial instance of collaborative spontaneous activity between children and parents in each interactions, analyzing interactions where both participated. Third, the analysis zoomed in on the longest and most sustained interactions between parent and child, conducting microanalytical assessments.

In analyzing the interactions of the parents and children, I used multimodal transcription with screenshots and images that follow Goodwin (2018) and Goodwin and Cekaite (2018). Central to this methodology is the “systematic investigation of the different kinds of semiotic resources and meaning-making practices that participants themselves attend to and treat as relevant as they build action within interaction together” (Streeck et al., 2011, p. 4). Through a combination of two open-source terminal-based programs, I employed ImageMagick (The ImageMagick Development Team, 2021) and FFMPEG (Tomar, 2006) to extract screenshots and anonymize them. I then edited the transcripts in an image editor as a part of the analysis (see also Chen, 2022a).

## 3 Results

### 3.1 Baseline phase

All three autistic children produced a variety of stims, which can be characterized as movement signatures: sustained postures and cycles of movement that are a common occurrence for a person (Streeck and Chen, 2023). These stims were all mentioned in the ethnographic interviews, and most were observed in the videos collected (see Table 2). Each of the children presented different sensory sensitivities, and always produced their movement signatures by themselves without the involvement of anyone else. During day 1 of the study, Matt would take a hard object, such as a plastic toy, and use it to tap repetitively on the ground. Nathan would swing by his door, playing with a shaker that he would keep in his hands. Chloe would play “Ode to Joy” on her phone again and again and produce repetitive humming.

**Table 2:** Sensory profiles of autistic participants.

	Touch sensitivities	Sound sensitivities	Frequent stims
Matt	Hypersensitive to touching others and heavily textured objects.	Hyposensitive to sound. Displays strong musical preferences towards piano sounds and certain nursery rhymes.	Produces haptic stims. Taps various surfaces (floor, boxes, table) with utensils and toys. Matt also jumps and flaps his hands.
Nathan	Hyposensitive to touch. Seeks out deep pressure massages and constant close proximity to his mother.	Neutral sensitivity to sound. Displays strong musical preferences towards piano sounds and certain nursery rhymes.	Produces vestibular stims. Sways, rocks, and swings frequently. Shakes noisy items continuously, such as shakers.
Chloe	Neutral sensitivity to touch, although she enjoys handling small objects like laminated pictures.	Neutral sensitivity to sound, with very strong preferences towards certain songs.	Produces musical stims. She plays songs like “Ode to joy” on the her mother’s phone again and again. She also repetitively vocalises and hums.

Day 1 revealed that all three families spent most of their time in the living room, with Matt and Nathan mostly under their large living room fan to escape Singapore’s heat. All three children also spent most of their time with their mothers, who therefore became the main interactants in the study. Matt would interact with his mother most frequently on the couch, sitting side-by-side. They would communicate with an alphabet board which he would point to and spell words out on. Nathan would interact with his mother most frequently on the floor. He often held onto her and pressed his body upon hers. Chloe would interact with her mother in the living room, jumping on her or running around her. She used her AAC device mostly for requests.

### 3.2 Intervention phase

After Day 1 of ethnographic interviews and collecting videos of the children’s everyday lives, Day 2 to Day 4 involved bringing the MMM into their homes. Once the research team had set up the cameras, and when two mats had been placed on the ground, the space was open to participants. The researchers moved aside and allowed the interactions to spontaneously unfold. Each of the parent–child dyads took some time to become acquainted with interacting with one another in the new environment. The artifacts augmented their living room floors, designating a physical area of novelty in their homes. This novelty was experienced both by the child and by the parent simultaneously, and thus both had to navigate the new environment together.

Although all three children had different sensory profiles and presented different relationships with their mothers, some consistencies were found across the dyads as they navigated the novel environment. In the following sub-sections, I discuss, (1) significant features of the children’s and parents’ first interactions on the mats and (2) the interactional transformation of the children’s stims during the intervention, constituting the most sustained interactions during the intervention.

#### 3.2.1 Children’s discovery of a novel sensory experience

When all the children first encountered the mats, they assumed a variety of initial postures. Matt and Nathan each laid down their mats, and Chloe knelt on hers, then got up and started running in small circles on it. Although all children initially assumed different postures, they all positioned their bodies within the boundaries of just one of the mats. The initial posture of all three parents when they encountered the new floorspace was similar: the parents assumed a sitting or squatting position on the adjacent mat, facing their children.

A striking similarity occurred in all the first interactions of the dyads: the children and their parents did not at first share the same agenda when they arrived at the mats. Consistently, parents pursued the project of having their children establish hand-to-hand touch, while children pursued the project of exploring the materiality of the mats and touching their parents’ bodies in their own way. Eventually, in their own time, all children discovered unique features of the environment that they then established a sensorial relationship with.

All the parents began recruiting hand-to-hand contact with their children when they first navigated social interaction on the MMM. The parents first extended their open palms to their children, creating a palm up open hand (PUOH) gesture (Ferré, 2012) that invited their children to touch their hand. All three children largely ignored the invitations from their parents. The parents then transformed their hand gesture into light-tapping upon their children’s thighs and knees. When the children did not react in any way observable to them, they then returned to extending an open palm to their children. The alternation between both offering an open palm, and producing light touches on their children’s bodies, were attempts to recruit them into participation, to perhaps spark his curiosity to interact more, and to achieve hand-to-hand contact with them.

A PUOH gesture heavily constrains the action possibilities of the child in several ways. Firstly, the child is expected to touch their parent—on a very specific area of the body (fingers and palm of the parent’s hand)—for interaction to happen. The child is therefore discouraged from exploring touch-based interaction with other parts of the body and exploring the environment in their own way. Secondly, touching a parent’s open palm presents a possible risk for control touch, wherein the parent’s fingers can enclose themselves around the hand or wrist of the child. Control touch by the parents—grabbing the children’s hands or wrists—also occasionally occurred in all the dyads. Children would remove their hands from their parents and turn away when control touch happened. All the children rarely produce an uptake to the PUOH invitation, largely ignoring their parents’ hands when the invitation presented itself.

**Figure 3:**
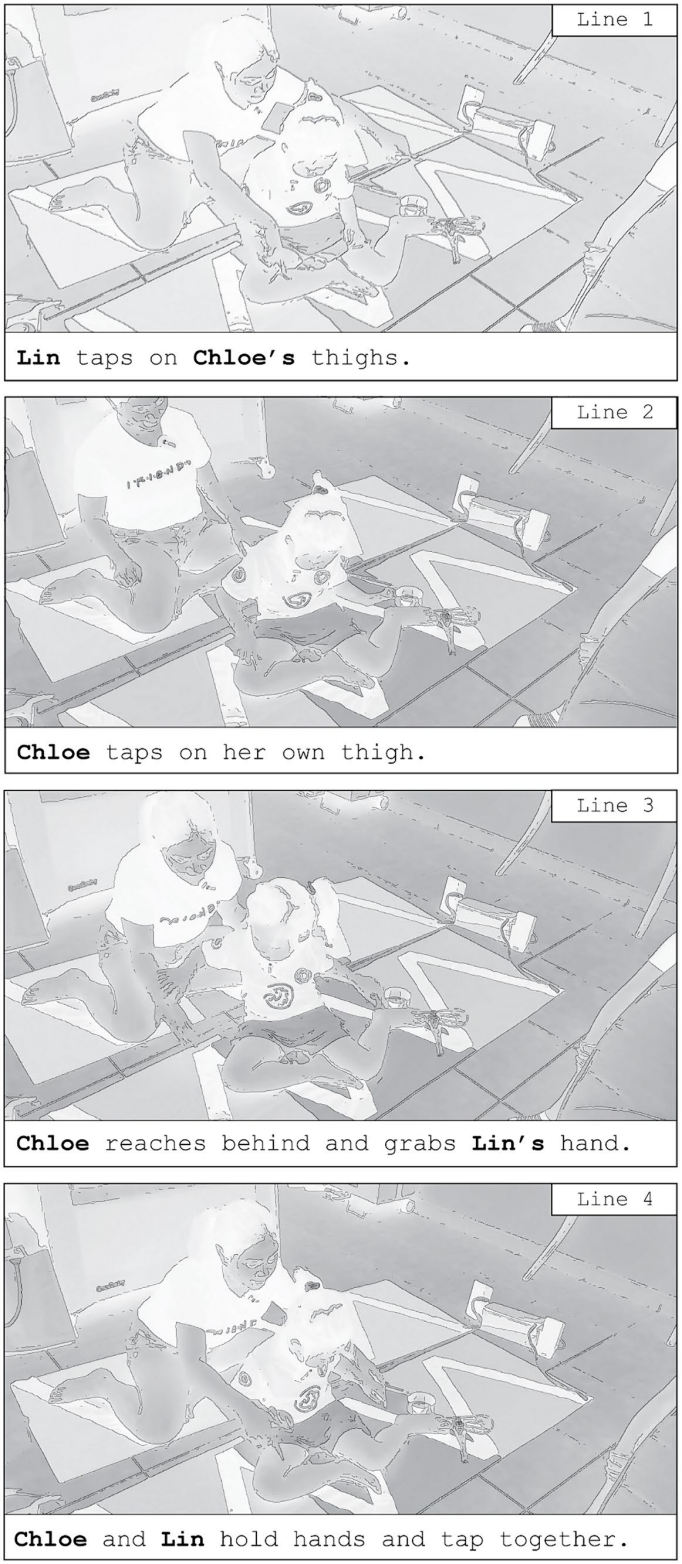
Chloe’s sequential discovery of the touch-to-sound feature of the mats.

Amidst these interactional constraints, all three children eventually found their own ways to discover sensory novelty in the environment. They all underwent the process of discovering the touch-to-sound feature of the mat while navigating conflicting agendas with their parents. As an example, the transcript in Figure 3 demonstrates Chloe’s discovery of the interpersonal touch to sound feature of the environment.

In line 1 of the interaction captured in Figure 3, Lin, seated behind Chloe, alternates between tapping Chloe’s right and left thighs with her fingers. Chloe looks down at her mother’s fingers upon her thigh. In line 2, Lin removes her hands from Chloe’s thighs and leans backwards. Chloe stares at her right thigh and taps it with her right hand. She does this action a few times with her right and left index fingers. Chloe then turns around slightly, reaching behind for her mother’s hands (line 3). She brings them forward above her thighs. In line 4, with Chloe’s hands on top of Lin’s, both Chloe and Lin hold hands as Lin touches Chloe’s thighs in alternation.

Chloe has to, in her own way, perform a series of actions that would lead her to the discovery of touch-to-sound. Chloe only discovers and appreciates the novel touch-to-sound feature/experience when she has time to explore and experiment. Matt and Nathan also undergo a process of experimentation, continuously asserting interactional effort to ignore their mothers’ invitations and to outrightly reject their control touch. It is only when they have sufficiently explored the environment that the children then seek out touch with their parent, coming to discover and appreciate the key design principle: mapping interpersonal touch to sound. The discovery process of interpersonal touch to sound is outlined as such:

1. Child and parent eventually arrive on MMM floormats.
2. Child realizes there is something novel about the environment.
3. Child produces ‘test’ touch actions on the mat, and on themselves.
4. Child touches parent and discovers touch-to-sound feature.
5. Child and parent begin co-constructing new interactional routines.

Through the learning process outlined above, all three children restructured their worldview of the environment (Nishizaka, 2006) by performing test actions on the artifacts, and coming to a new understanding about core features of them. After this discovery process, they perform actions that display sensitivity to their new achieved structure: during interactional sequences after, they return to the touch-to-sound interaction and experiment with the sound even further. Despite the strong agendas of their parents, the children assert their autonomy and explore the novel environment at their own pace, in their own ways.

#### 3.2.2 The interactional transformation of stimming

Although their invitations to engage in touch sometimes involve taking objects away from the children, performing control touch actions upon the children’s hands, or misinterpreting the action trajectories of the children, parents eventually found creative ways to come ‘in touch’ with their children: by facilitating the enactment of a pleasurable sensory experience for the child. Interpersonal touch, being a core design feature of the augmented environment, eventually oriented all the parents towards objects and surfaces their children were interacting with by hand.

Parents brought objects onto the mat and used them in creative ways with their children. Sustained, mutually elaborative interactions often occurred when parents attended to the stimming of their children, whether through engaging in tapping, swinging, or playing the same piece of music together. These old patterns and routines of the children—stimming—were always solitary and did not tend to involve others. In the new circumstance of the intervention, however, new forms of relating emerged when parents joined in the stimming routines of their children, facilitating their production, and transforming them into new shared experiences.

##### 3.2.2.1 Turn-taking in tapping together

The most sustained interactions between Matt and his mother, Danna, occurred when the researchers moved the mats to the couch, a physical area within which the dyad frequently interacted with one another. In their everyday lives, Matt and Danna would often sit side-by-side on the couch in the afternoons, where they would read books, converse, and watch television together. During Day 2, and after several interactions of navigating his mother’s control touch over the movement of his hands, Matt left the mats and headed to another part of the living room to tap by himself. He ignored his mother’s multiple verbal invitations to return to the mats. As an attempt to facilitate more interaction, the researchers moved the mats to the couch, so that both participants could sit on the couch with their bare feet on the mats. Danna asked the researchers how the mat worked, which allowed her to realize that music could also be produced by being haptically linked to Matt through a conductive object. Knowing that Matt enjoys tapping, Danna then brought some metal utensils—four spoons—onto the couch with her. She sat on the couch with her feet touching one mat, inviting Matt to sit down beside her.

In their unfolding interaction, Matt takes up Danna’s offer to tap upon her spoon. Once he begins tapping, the spoon gathers momentum by springing up and down with some buoyancy. With each hit, the metal spoons emit a clanging sound, embellished with 2–3 musical notes from the floormats. Matt’s experience of hitting Danna’s spoon with his spoon thus provides haptic feedback up his arm, but also becomes a different sonic experience than the usual items he taps. The taps are more percussive: the clanging is crisp and resonant, and the musical notes make the impact of Matt’s taps on Danna’s spoons more prominent. Each time he taps on Danna’s spoons, the musical notes are different.

**Figure 4:**
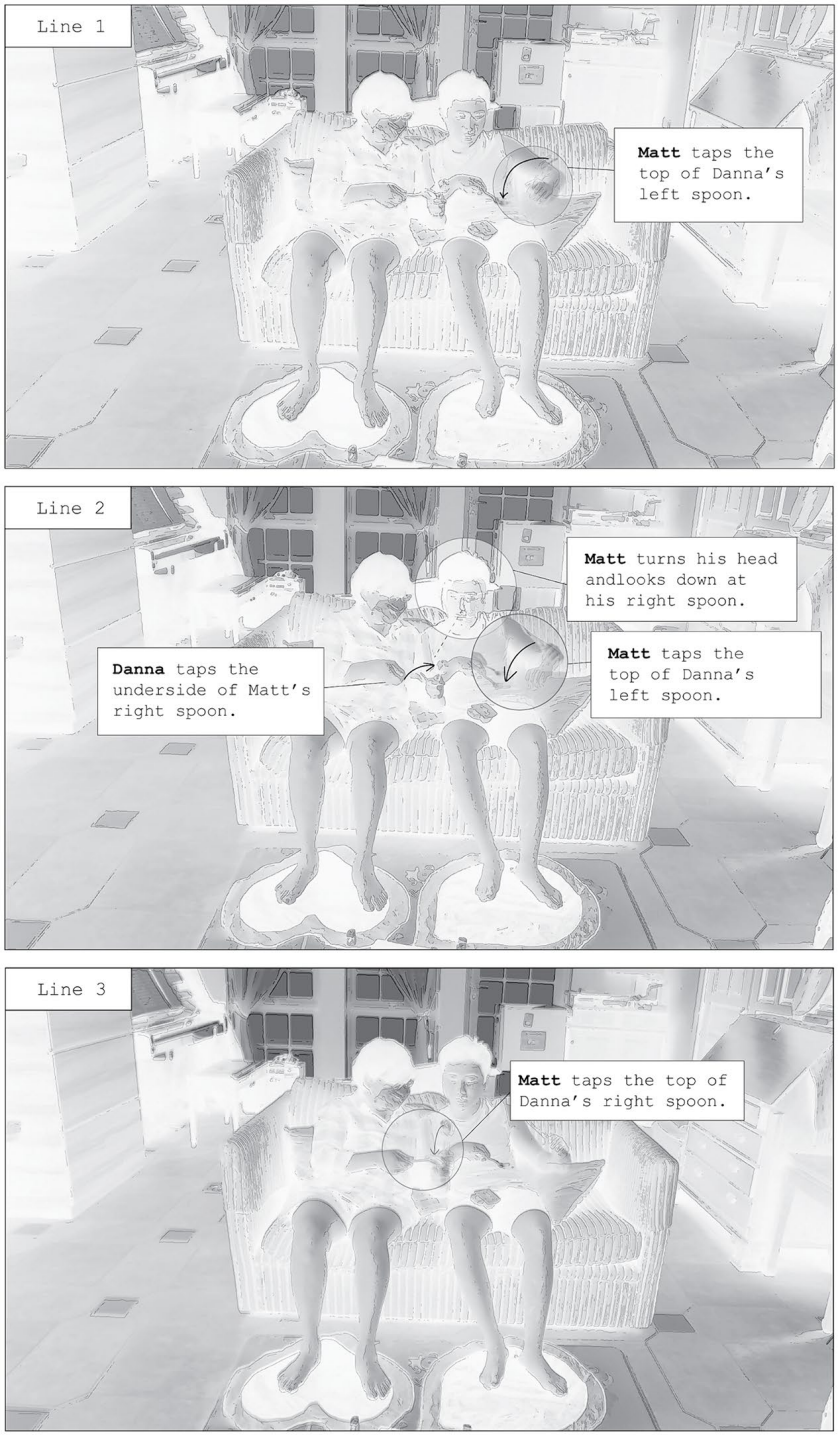
Matt and Dana’s turn-taking sequence.

As Figure 4 demonstrates, when Matt joins his mother (Danna) on the couch, she offers him two spoons. Matt and Danna hold a spoon in each hand, and Danna then slips her left hand through the gap between his forearm and the side of his body. With their forearms intertwined, Danna raises the spoon in her left hand towards the spoon in Matt’s left hand. Danna’s action offers her spoon as a surface for Matt to tap upon. Unlike the PUOH gesture, which poses the possibility that Danna close her fingers around Matt’s spoon, Danna’s spoon is a material surface that cannot perform control touch upon Matt’s spoon. Matt takes up the offer, repetitively using the spoon in his left hand to hit her spoon (line 1), creating a loud ‘clang’ each time they hit utensils. Tinkly piano sounds also play each time Matt’s and Danna’s utensils touch one another. After a few taps, Danna raises her right hand towards Matt’s right hand (line 2). Matt’s eye gaze shifts from his left hand to his right hand, and for a moment, he glances at the spoon in his mother’s hand. Matt begins to tap his mother’s spoon with his right hand, pausing the tapping in his left hand (line 3). After a few cycles of tapping upon Danna’s spoon, Matt encounters another invitation from Danna to tap upon her left spoon with his left hand. Matt responds by tapping with his left hand, and thus, enters into a repetitive cycle that is different from his usual tapping. He alternates between tapping with his left and right hands, alternating between the taps even during later cycles, without Danna’s invitations. These alternating taps are also simultaneously felt by Danna as her spoons are tapped upon. She ensures that the spoons are close enough to Matt’s hands so that he can continue tapping on them with ease. After a few rounds of this new repetitive cycle, Matt begins to explore larger stim movements: he extends his spoon at varying distances from Danna’s spoon, creating different sounds as he brings his hand down to tap. Stronger taps create quicker and higher pitched musical sounds, and lighter taps create slower and lower pitched musical sounds. A few rounds after, Matt holds his spoon still, and Danna begins to tap upon it. Both parent and child take turns in tapping and offering their spoon to be tapped upon.

For Matt and Dana, Matt’s old patterns of tapping are transformed into new repetitive cycles, established conjointly by both. When Danna facilitates the enaction of Matt’s stims, they are transformed in three ways. Firstly, Matt’s stims are no longer produced by just one hand, but at this point involve an alternation between both his hands. Secondly, Matt’s stims now require the spoons of his mother in order to be produced with haptic feedback to his hand, and with the loud sounds that always occur with his taps. Thirdly, Matt explores new stim actions by varying his own tapping motion, playing with the distance and force of his taps upon his mother’s spoon. In addition, these new co-created repetitive cycles allow Matt to include his own creative actions: he plays a part in the turn-taking, providing his spoon for his mother to tap. Through fine attunement to the facilitation of Matt’s stims, Danna co-creates a new sensory experience with Matt, one that allows him to continue his tapping but in a way that involves her.

##### 3.2.2.2 Sensorially transforming swinging together

As with Matt and Danna, Nathan’s and his mother Ellie’s most sustained interactions also involved the transformation of Nathan’s stims. Nathan, as observed during the researcher team’s first visit and as reported by his mother, would often sit in his swing and rock himself. The swing itself was in a large, designated area, occupying much of the physical space by the family’s front door. Although Day 2 did not yield any significant transformations of Nathan’s stims, Day 3 and Day 4 did. During our third visit to Nathan’s home, Nathan left the living room floor space and spent some time on the swing. The research team suggested putting the mat on the swing and another on the ground. We waited for Nathan’s swings to stop before we slowly approached him and gently placed a mat under him. We placed another mat on the ground right in front of the swing, establishing a spot for Ellie to stand on and another for Nathan to continue sitting on. Nathan had brought a hairtie onto the swing with him: it was the hairtie of one of the research assistants. He twiddled with it as he swung.

With Nathan sitting on the swing and Ellie standing in front of him, Ellie begins to recruit him into interaction with her. She stands on the mat, extending her right palm towards Nathan. When Nathan does not respond to the open palm invitation, Ellie gently holds his fingers, and begins to pull and push on them. Her pushing and pulling rocks Nathan back and forth, but not for long. Nathan removes his fingers from her grip and turns his body away from her. Rachel then makes a comment, stating “you touch feet also can,” meaning that music would play if both touched feet. Nathan begins to produce slight kicks with his feet as he swings towards Ellie, his toes creating piano sounds as they touch the front of his mother’s ankles. As demonstrated in the previous section, Nathan does not produce any uptake to the open palm invitation from his mother. Nathan also rejects his mother’s control over the movement of the swing, removing his fingers from her grip and turning away from her. Exercising agency, his first contact with Ellie is through light kicks upon her shins, which produce musical sounds. After a few swing cycles of light kicks, Ellie extends her hand, placing it close to Nathan’s hands. Nathan passes Ellie the hairtie, and she puts it up her arm. The following interaction ensues.

**Figure 5:**
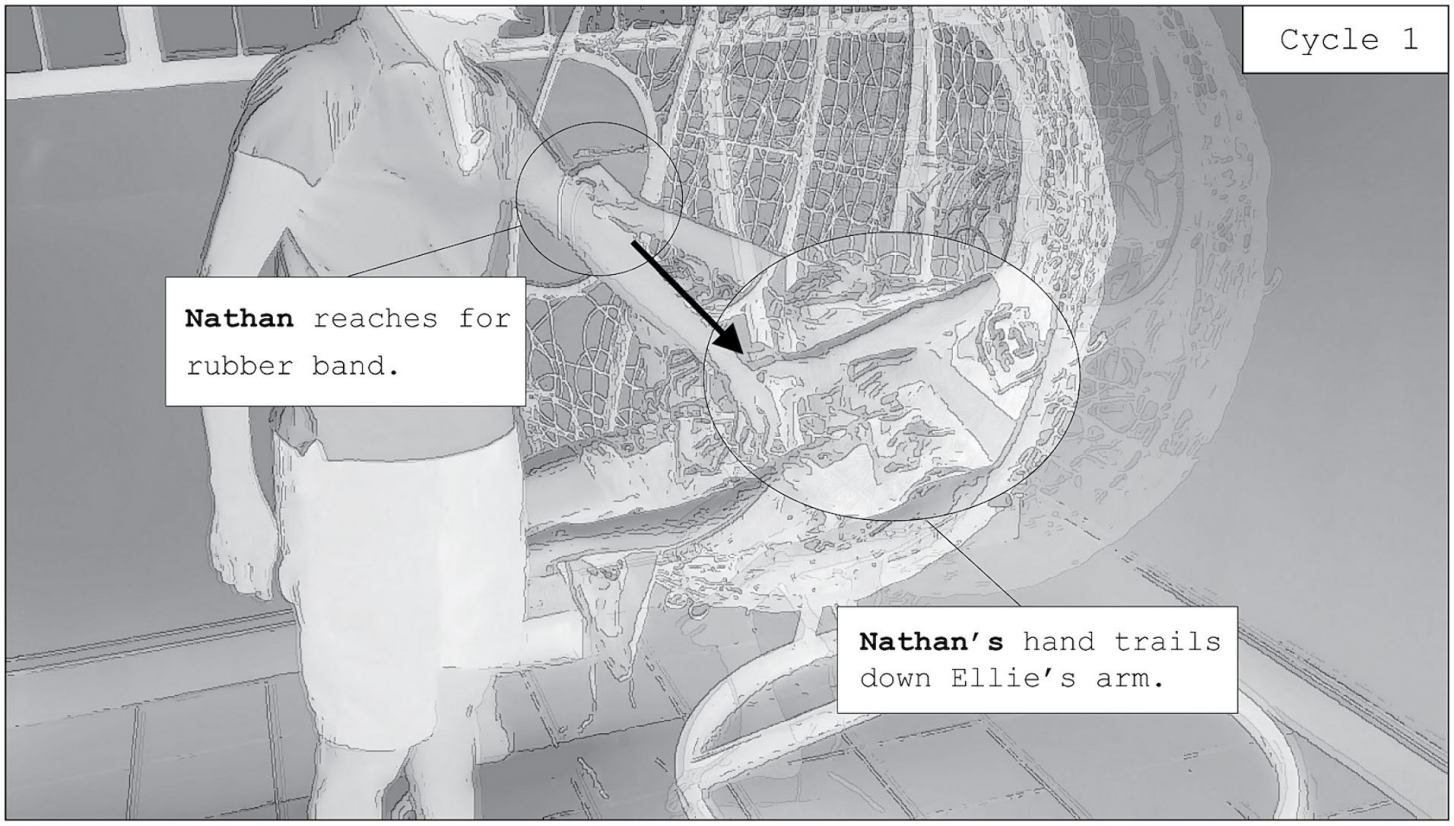
Nathan and Ellie begin cycle 1 of a novel interaction on the swing. Two screenshots are overlayed to demonstrate the trajectory of Nathan’s hand.

In Figure 5, as Nathan swings forward, he reaches his right hand out towards the hairtie on Ellie’s arm. He does not lean forward but remains nestled against the soft pillows on the swing. Nathan allows the momentum of the swing to carry his body forward, making contact with Ellie’s arm lightly, but not trying too hard to grab the hairtie. Once he reaches the apex of his swing forward, he touches the hairtie and lets the tip of his fingers sweep down the length of Ellie’s arm as he swings backwards. The mat produces a series of piano sounds that gently trail off when he loses touch with Ellie’s skin as the swing moves backwards. Several cycles of this interaction occur, with Nathan swinging forward and reaching for the hairtie. During these cycles he misses grabbing the hairtie, but maintains the stretch of his hand, lingering his fingers on Ellie’s arm until he swings out of reach. Each swing away from Ellie is thus augmented with a series of piano sounds. These cycles are repeated several times before it evolves.

**Figure 6:**
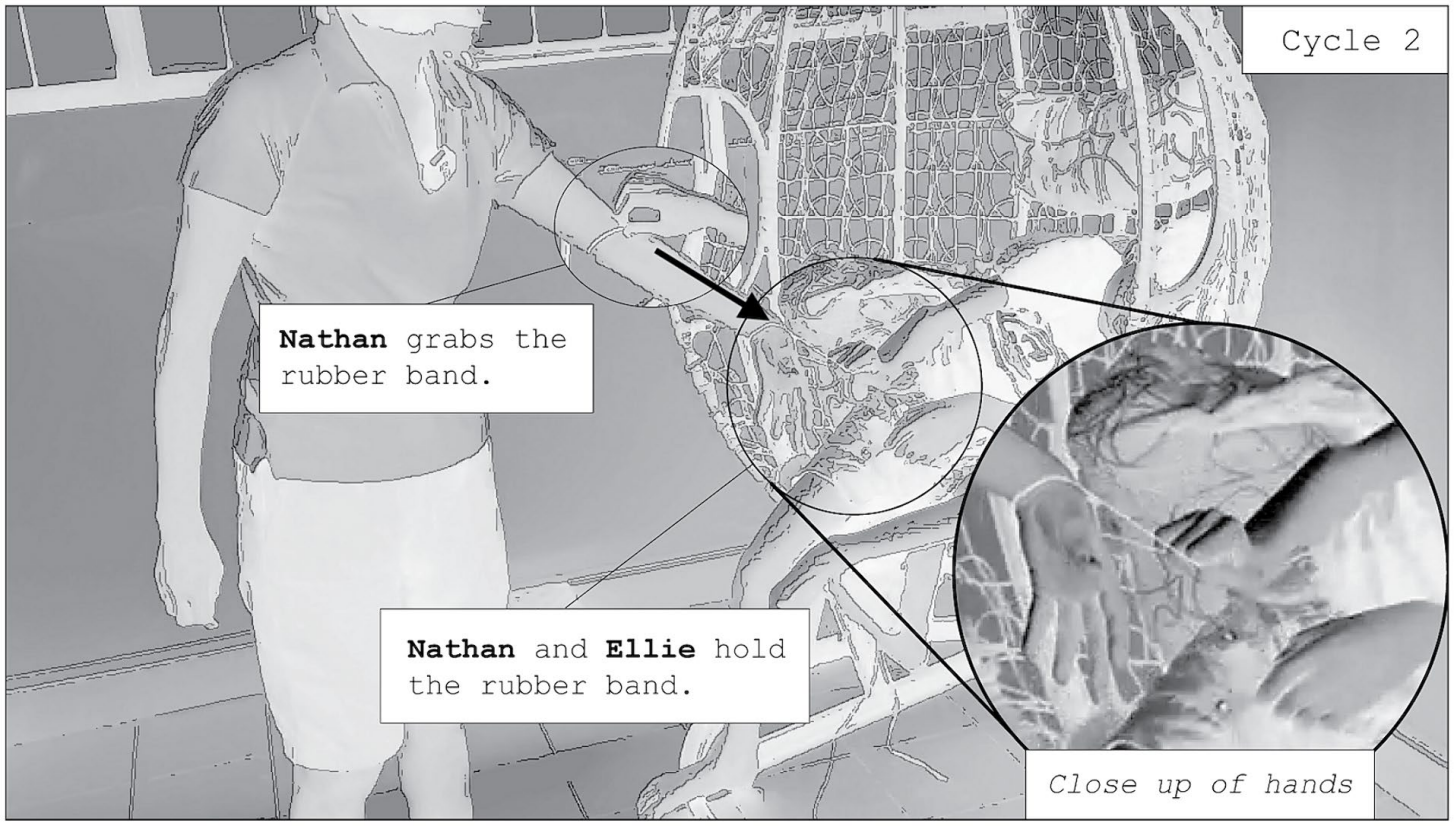
Nathan and Ellie begin cycle 2—the next evolution—of their interaction on the swing. Two screenshots are overlayed to demonstrate the trajectory of Nathan’s hand.

Nathan eventually grabs the hairtie on his mother’s arm (Figure 6). He does so by putting his thumb under the elastic and curling his index finger around the material. As he swings backwards, Nathan maintains his hold on the hairtie, such that it is brought down to Ellie’s wrist. She points her fingers downwards, preventing the hairtie from leaving her wrist as Nathan swings backwards. With both Nathan and Ellie haptically connected by the tension of the hairtie, the interaction evolves into another repetitive cycle, and Nathan’s swinging movement is further transformed. As Nathan swings back and forth, he is now pulled back to-and-from Ellie through the tension in the hairtie. Nathan makes hand-to-hand contact with Ellie each time he swings forwards, and the mat produces a quicker, percussive series of piano sounds each time this happens. The periodicity of Nathan’s swings is thus sonically amplified, with sound punctuating the swing cycle only at the apex of his swings. Nathan laminates further action onto their interaction in later repetitive cycles, continuing to touch Ellie’s hand, but also touching her ankle through slight kicks of his leg when he is close enough to her. As more repetitive cycles unfold, Nathan eventually takes the mat from under him, and begins to crinkle it as he continues to hold onto the hairtie and touch his mother’s hand, enhancing his sensory experience even further.

Nathan’s interactions with his mother involve repetitive cycles of swinging back and forth. Through these cycles, the interaction gradually evolves, with Ellie facilitating the momentum of the swinging through the tension of the elastic band held between her hand and Nathan’s. This interaction is sustained and mutually elaborative because Ellie does not control the momentum of Nathan’s swings. She allows him to be in control of his swinging, her social actions serving only to auditorily and haptically enhance his ongoing repetitive movement. Nathan himself participates in the interaction, evolving their interaction and gradually elevating his unfolding sensory experience. First, he touches Ellie’s forearm as he swings, sonically amplifying the trajectory of each swing backwards (cycle 1). Next, he grabs the hairtie, using its tension to produce quicker swings towards her. He then laminates further action upon the interaction, kicking her legs and adding more musical sounds. Eventually, he removes the mat from under him and instead places it on his lap, crinkling its surface with one of his hands, adding to his ongoing sensory experience. Nathan transforms his swinging experience accumulatively, amplifying his sensory experience to include a variety of sounds, changes to the motion of his swinging, and crinkling the mat as he swings. Nathan’s usual swinging practice, typically produced by himself, now becomes an amplified sensory experience that is shared with his mother. They establish new swinging cycles that collaboratively evolve over time.

##### 3.2.2.3 Playing “Ode to Joy” together

Chloe and her mother, Lin, also engage in multiple co-constructed repetitive cycles, as did the other two dyads. Chloe had a strong preference for a particularly musical stim; she would often play the song “Ode to Joy” again and again on a piano app on her mother’s phone. Noting this repetitive practice, “Ode to Joy” was set up in the Magical Musical Mat, such that every finger movement would trigger each consequent note of the song. Three different timbres were programmed into the mat, so that different degrees of skin-to-skin contact allowed for different types of sounds to play. Day 2 of the intervention already yielded sustained stimming between Chloe and Lin.

**Figure 7:**
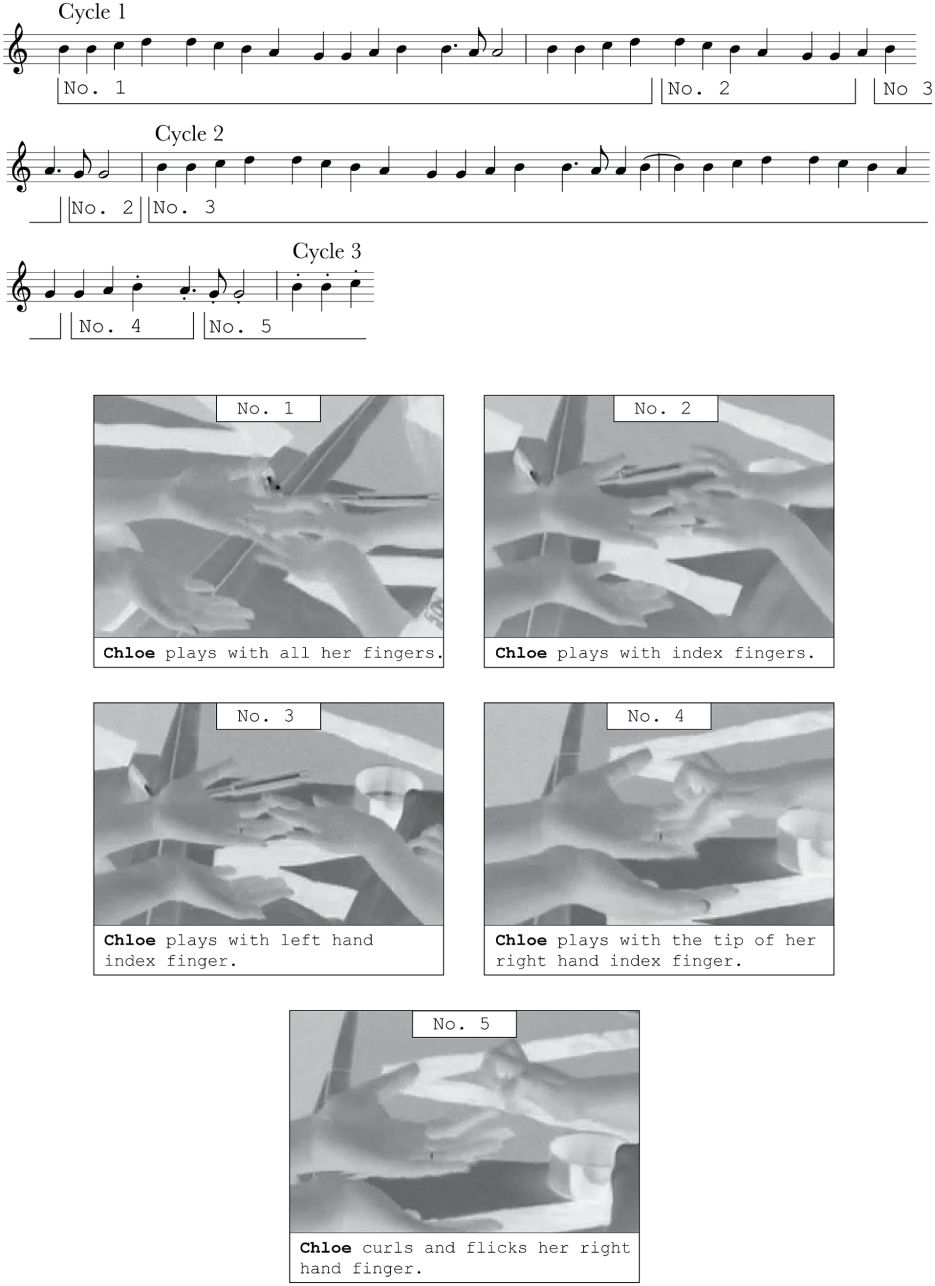
Chloe plays two cycles of “Ode to Joy” on Lin’s hands. Each cycle denotes a full sequence of the tune “Ode to Joy.”.

In their most sustained interaction, Lin successfully recruits Chloe into interaction with her by sitting on one mat, inviting her to sit on the other. Lin extends her fingers towards Chloe, not with her palm up, but rather with her palm down. She says “piano” with a sing-song prosody, and Chloe begins to play on her mother’s hands as if her mother were a piano. Round upon round of playing “Ode to Joy,” Chloe explores a variety of touches upon her mother’s hand, keeping a consistent tempo to her playing, but changing only the way in which she touches her mother. Figure 7 details Chloe’s production of “Ode to Joy” over two cycles of the song, and the unfolding of her varied, exploratory touching of Lin’s hands.

As Chloe plays through cycles of “Ode to Joy,” no cycle is played in the same way. Chloe varies the way in which her hands interact with Lin’s, performing a range of touch-based gestures upon her mother’s fingers. As seen in Figure 7, over the course of cycle 1 of “Ode to Joy,” Chloe uses all her fingers to gently pat the fingers of her mother (No. 1). The mat plays “Ode to Joy,” with a slightly sustained note each time. During the second half of cycle 1, Chloe changes the shape of her hands slightly. She holds her wrists up, touching Lin’s hands with only the very tips of her index and middle fingers (No. 2). Chloe uses this hand shape for just a few notes of the piece before trying yet another hand position. She lowers her right hand and plays upon Lin’s fingers with her left hand index finger (No. 3). The decrease in skin contact with Lin results in two notes of “Ode to Joy” played with staccato: notes of very short length. Without missing a beat, Chloe plays the last two notes of cycle 1 with the index fingers of both hands, such that the song ends off with two sustained notes. Chloe thus follows the musical structure of “Ode to Joy” by ending it with two long notes (following the original song), providing a more complete close to cycle 1.

Chloe continues with the momentum of the music without missing a beat, performing a variety of gestures that change the quality of the sound playing from the mat. Eventually, Chloe explores even more touch-based gestures, curling her fingers, and touching different parts of her mother’s hands. In cycle 2, Chloe returns to the gesture depicted in No. 3. She uses just a bit more skin contact that previously, so her use of one finger does not trigger any sound changes, and Chloe continues with this gesture for most of cycle 2. At the end of cycle 2, Chloe makes another change to her gesture production. She switches from using her left hand to using her right, touching the tip of Lin’s fingers with the tip of her fingers. The decreased skin contact with Lin begins to trigger a staccato sound in the mat after two notes with this gesture. To decrease her skin contact with Lin’s finger even further, Chloe begins to touch the tips of Lin’s fingers by curling and flicking her index finger towards her palm (No. 5). Consistently, she continues with the staccato sound for the next cycle, then continues to produce more variation to her touch-based gestures over the course of the next few cycles. Throughout the rest of the intervention, as Chloe and Lin cycle through “Ode to Joy,” the interactions evolve. Chloe explores diverse finger movements on Lin’s hands, attuning to the musical structure of the song in the sounds she produces. In later repetitive cycles, Chloe and Lin move on to explore different ways of touching one another, whether through tapping on Chloe’s thighs, holding hands and swaying together, or cuddling together.

Chloe, who usually plays “Ode to Joy” on her phone by herself is now able to articulate individual notes of the song upon Lin’s body, nuancing the quality of each note. “Ode to Joy” becomes an act of self-expression, but also one that she can share with her mother within these particular circumstances. Chloe and her mother also move beyond the hand-to-hand interactions and experience “Ode to Joy” through other parts of their bodies. As with other dyads, Chloe and Lin transform Chloe’s solitary practice of playing “Ode to Joy” into a new shared experience of musical nuance, spontaneity, and expression.

## 4 Discussion

The results underscore significant transformations in parent–child interactions. Upon encountering the novel artifacts, parents and children alike demonstrate curiosity. Nevertheless, their initial interactions are built upon their bodies’ relationship history. At first, parents extend invitations for hand-to-hand touch, attempting to engage their children in the new environment, at times even resorting to control touch. However, the children assert their autonomy and explore the novel environment at their own pace, discovering unique sensorial features of the environment. Notably, the foregrounding of interpersonal touch guides parents into their children’s sensory activities. The most sustained, mutually elaborative interactions involve parents attuning to the stims of their children by facilitating their expressiveness, thus co-creating a pleasurable sensory experience together. The introduction of sound prompts heightened awareness of the children’s stims, leading to diversified and expressive stim movements whether through tapping, swinging, or playing “Ode to Joy” together. All three children add ‘embellishments’ to the rhythmicity of their repetitive behaviors, increasing the musicality of their stims. All the parents and children eventually co-construct longer patterns of repetitive cycles together, laminating action upon action, with the cycles evolving over time.

The stimming practice of the children, different as they are, all become substrates for interactional reuse and transformation. A substrate is an utterance or any other public resource that serves as a point of departure for operations used to build subsequent action (Goodwin, 2018; p. 40). The substrate—stimming movements, in this case—thus becomes a mutually agreeable focus of transformative operations for both the autistic child and their interactant. With each cycle of the stims—each tap, each swing, each finger movement—sonically amplified by touch with their parents, musicality becomes an additional medium that the children can play with. In Matt’s case, from his usual practice of tapping with just his right hand, he comes to alternate between tapping with his left and his right hands. In other parts of his tapping interactions, he also takes turns with his mother in tapping and being tapped upon. For Nathan, the push and pull of his swing become part of a game involving reaching for the hairtie on Ellie’s arm. When he eventually grabs it, and pulls it downwards, his swinging movement itself becomes incorporated into the tension of the hairtie that is being grasped by both. Much like a pendulum, Nathan swings back and forth, with the swing cycles evolving over time, no swing ever the same. Lastly, Lin’s hands are transformed into the keys of a piano (or perhaps a touch-based synthesizer) that Chloe can play her favorite song upon in a musically expressive manner. The parents Danna, Ellie, and Lin, by coming into intercorporeal attunement with their children’s stims, become a central part of the stimming practice, and also get to experience its motion upon their own bodies.

Furthermore, the periodicity of the children’s stimming movement is amplified with musical sounds, bringing into activity the creation of a new sensory experience, amplifying the presence of their stims. The children’s stim movements change, become more varied and expressive, and the children embellish the periodicity of their repetitive behaviors. Matt varies the height of his arm, dynamically changing the volume of the taps he produces, varying the assertion of his spoon hitting upon Danna’s in a way that creates different musical pitches. Nathan creates rhythmic variations of his own by touching his mother’s forearm and hand, followed by the peppering of little kicks upon Ellie’s shin. These additional movements laminate musical sounds upon his swinging motion. Chloe plays with a variety of timbral changes upon her mother’s hands, matching them to the musical structure of “Ode to Joy.”

By coming into touch with one another, the solitary stimming practices of the autistic children now require the participation of the other. These stimming movements become part of a larger social ecology, changing in how they are being produced. The interactional cycles between parent and child can be likened to the cultural practice of free improvisation. As Duby (2020) posits, free improvisation, in which participants interact in an unscripted manner, is ‘at the edge of chaos’ (p. 8) by operating in a state that is far from equilibrium. The process of improvising together requires a dynamical coherence between individuals through a blend of stability and active flexibility (Laroche and Kaddouch, 2014). Each dyad came to create and kept creating repeated cycles that stable and predictable. Yet, no two cycles were ever the same, and anything that was repetitive was not for long. As time went on, each of the dyads’ interactions evolved co-operatively (Goodwin, 2018), where material from a previous cycle became material for the next, in cycles of reuse and transformation.

Despite the study’s rigor, several limitations affect the interpretation and generalizability of findings. Firstly, due to the paper’s limited scope, further analyses of interactive stimming were restricted. Whereas sustained sequences of interactive stimming examined, briefer bursts—encompassing a range of stimming practices—could not be addressed in this paper. Additionally, the trajectory of interactive stimming over the intervention period could not be thoroughly explored, impeding the identification of overarching patterns in the dyads’ evolving stimming practices. Furthermore, there was insufficient space to include family comments from the debriefing session, which would have provided valuable contextual insights to the study.

Secondly, the study is limited in its participant selection. The study’s size, involving just three autistic individuals and their families, impacts the generalizability and depth of analysis of the results. The broad age range of autistic participants introduces developmental variability that may confound the interpretation of the findings. Lastly, the study lacked a follow-up evaluation to determine the long-term efficacy of the intervention on communication practices.

Addressing these limitations is crucial for advancing an understanding of interactive stimming among autistic individuals. Future papers reporting on this intervention could include the debriefing interviews conducted at the end of the study, account for all sequences of interactive stimming, as well as analyze the longer trajectory of interactive stimming that occurred over the course of the intervention. Future studies should consider employing longitudinal designs to thoroughly investigate the routinization of interactive stimming and the effectiveness of interventions that encourage its development. These approaches will provide a more comprehensive understanding of how interactive stimming patterns evolve over time and how various interventions influence their emergence.

## 5 Conclusion

To experience a fulfilling social existence as a human being is to be recognized by others as having a rich cognitive life, and to produce actions that are taken up by others as building a longer trajectory of action together (see also Goodwin, 2004). The interactions between autistic and non-autistic interlocutors have been forefronted as the locus of the ‘double empathy problem’ (Milton, 2012), with the more vulnerable communicator bearing the consequences of interactional disjunctures. But what does it mean to truly practice empathy as the more privileged interactant?

As stated by Allen (2021), a non-speaking autistic writer, the presumption of competence—a principle of respect—by speaking interactants is not an act that can be completed. It is a constant work-in-progress by speaking others that requires making mistakes and learning from them, especially when in daily life the flow of verbal conversation is maintained and dominates the interactional space. Stepping beyond the binary of ‘speaking’ and ‘non-speaking’ is to first understand sociality as interactional practices that occur through interweaving a multitude of modalities (see also Sequenzia and Grace, 2015; Savarese, 2021) and being open to “an opportunity to make the strange familiar” (Maynard and Turowetz, 2022; p. 60) through becoming aware of social actions that easily go unnoticed. Empathizing with a differently disposed social actor is about opening oneself to transformation through contemplating dimensions of experience that are different from one’s own (Liberman, 1999; Chen, 2021; Streeck and Chen, 2023), and thus arriving at a new commonsense (see also Maynard and Turowetz, 2022).

Given the contrasts between autistic and neurotypical minds, achieving a common experience can hardly be possible. A crucial consideration is how Autism constitutes its own unique culture: identical cultural material, such as music, can yield profoundly different results with autistic and neurotypical individuals (Straus, 2013). Distinctive cognitive styles—systems of thinking, making, and experiencing—can thus interact with cultural material in different ways (Bakan, 2018; Nitzberg and Bakan, 2021). As a neurotypical interlocutor, coming into attunement with an autistic mind involves stepping beyond one’s own state of being, taking up others’ actions as valid compositions, and coming into a shared world of perception and action that allows people to build new actions together. These new routines can happen if neurotypical minds seek to understand and participate in rhythmic routines different from one’s own. In this study, three different non-speaking autistic children and their respective parents navigate interaction together in an augmented living room space and learn how to interact with each other in new ways. By paying close attention to the embodied interactions of non-speaking autistic individuals, this study surfaces: (1) their diverse social actions; and, as such, (2) opportunities for social interaction with speaking non-autistic others.

Cultural tools are embodiments of the function and meaning of sociocultural practices, where learners actively reconstruct the tool’s normative meaning and function, through acting upon and with them (Saxe, 1994). The design and development of artifacts, tools, and environments has the power to transform daily communicative practices on both small and large scales in a multitude of ways. This study features an interactive environment that was an effort to bridge the gap between two divergent communicators. Firstly, bodies were brought together in close proximity, wherein intercorporeal attunement through touch—one of the most foundational ways to connect with another—was brought to the fore. Secondly, music created a cultural bridge, being a common medium of enjoyment for both parent and child. The MMM augments not the more vulnerable communicator, but rather their environment, as an attempt to surface more inclusive interactions. Through forefronting interpersonal touch and music, the MMM frees interlocutors from the instinct to maintain verbal conversation, temporarily suspending the rules of normative interaction. The MMM creates a situation where coming into intimate, multisensorial interaction is sanctioned and encouraged. But the MMM is merely a trick. In the folk tale of the stone soup, a magical stone is placed in boiling water for the creation of delicious soup. As time continues, additional ingredients—vegetables, meat, and other condiments—are added to the soup gradually. When the soup is done, the stone is taken out, and the nourishing food is enjoyed. In a similar way, the MMM became the stone soup of each home, where additional objects were brought into the space, and interaction occurred accumulatively and gradually. These interactions are available all the time, with or without the magic of the mat, should participants wish to engage as such, and should parents notice and recognize the social actions of their children.

Some theoretical, methodological, and practical implications are borne from this study. Firstly, that stimming can occur between autistic children and their parents invites a fundamental rethinking of the diagnostic criterion for autism, which characterizes solitary stimming and a ‘deficit in social communication’ as two independent hallmarks of Autism. This study, and more broadly the phenomenon of interactive stimming, pulls the two diagnostic attributes of Autism into question: the manifestation of repetitive behaviors can in fact flourish autistic communication. Interactive stimming also poses a tension to the common conceptualization of stimming as periodic self-regulation. There is no doubt that stimming, to a large degree, is a core facet of autistic embodiment in regulating emotions, regulating sensory input, and regulating sensory pleasure. However, emotional regulation is an act of socialization from infancy (Hertenstein and Campos, 2001), where family members all partake in soothing bodies in distress (Gross, 2007; Thompson and Meyer, 2007; Goodwin and Cekaite, 2018) Interactive stimming demonstrates that every human action occurs in a social world, where individual expression can diminish or prosper in the face of others.

Secondly, the methodological approach exemplifies the value of deep, qualitative analysis without the prescription of pre-defined labels and variables. For a population especially attuned to the sensory properties of people and artifacts, it is only through microanalyses that their nuanced interactions and co-created sensory experiences can be surfaced. Through the process of careful transcription, otherwise unnoticeable social actions were made visible. Furthermore, there is great importance in examining the researcher’s participant roles during data collection, especially when research involves bringing artifacts into the environment that are meant to enact some change. Given that the researcher is in a position of power when working with disabled individuals, microanalyses also allow for research practice to be increasingly oriented to the wellbeing of the most vulnerable participant throughout the study.

Thirdly, this study has practical implications for how parents, therapists, and educators can approach, understand, and support communication with non-speaking autistic children. The study proposes that inclusive education with neurodiverse individuals begins by embracing and enhancing their diverse interactional practices, so that their multisensorial social actions can be noticed and recognized as such. It joins a larger paradigm shift in designing for disability, towards moving beyond the individual and making physical environments more inclusive (Hart, 2014), marrying function with aesthetics (Pullin, 2009), as well as the idea of universal design for learning (Rose, 2000; Tancredi et al., 2022), where designing for multiple ways of learning, interacting, and being benefits not just those who are disabled, but everyone. This study proposes a reconceptualization of the AAC interface. By going beyond the screen and forefronting the communicative potential of the body, AAC systems have the potential to lower multiple barriers for the social participation of autistic individuals by embracing their naturally-occurring communicative practices.

Lastly, embracing musical expression has potential to open doors for communication, healing, and learning. The detailed analyses of these interactions reveal musicality as a dimension to be analyzed in social interaction, where participants engage in acoustic rhythm, melody, and timbre. Adopting a musical ear on phenomena such as stimming and repetitive behavior more broadly opens an abundance of analytical possibilities for the study of human cognition, expression, and interaction.

This study shows how stimming can become even more expressive when mapped with touch and sound. Perhaps more importantly, all the stims reported include the production of sound, even when they occurred naturally. The Magical Musical Mat demonstrates the potential of designing for sound interactivity: making careful design decisions around sound sensitivity, aligning with the sonic preferences of non-speaking autistic individuals, and creating environments in which they can themselves create new sonic material with others, can more broadly inform research on sound perception and our human relationship with music. Despite its promise, some caution is required around the utility of music. Music remains a culturally-sanctioned medium by those who are non-autistic, but nonetheless constitutes a widely accepted convention. Stimming should be embraced, even outside of the musical context.

The autistic mode of engagement, existing in an often unforgiving world, is fundamental to our very essence of humanity. In deep existential vulnerability, the permeability of autistic individuals with the world unveils human practices fundamental to human *being* (Fein, 2018), including a particularly embodied and sensory relationship with materiality and others (Baggs, 2007; Yergeau, 2010). As discussions on inclusion, diversity, and equity gain prominence, non-speaking autistic individuals unearth the foundational role of the body in all aspects of daily life, offering lessons on sensing the forgotten. It is my aspiration that researchers engaging with vulnerable populations actively incorporate the perspectives of disabled individuals, demonstrating a dedication to openness, change, and transformation in their work. Together, these considerations only strengthen our capacity for bridging research and practice, and for bridging the gap between diverse communicators.

## Data availability statement

The anonymized raw data supporting the conclusions of this article will be made available by the authors, without undue reservation.

## Ethics statement

The studies involving humans were approved by University of California, Berkeley. The studies were conducted in accordance with the local legislation and institutional requirements. Written informed consent for participation in this study was provided by the participants’ legal guardians/next of kin. Written informed consent was obtained from the individual(s), and minor(s)’ legal guardian/next of kin, for the publication of any potentially identifiable images or data included in this article. Nonessential identifying details were omitted from the data. Pseudonyms were given to participants, and screenshots were converted to line drawings for anonymization.

## Author contributions

RC: Conceptualization, Data curation, Formal analysis, Funding acquisition, Investigation, Methodology, Project administration, Resources, Software, Supervision, Validation, Visualization, Writing – original draft, Writing – review & editing.

## Footnotes

^1^In this paper, I follow the preferences of the Autistic community for “identity-first language” (e.g., Autistic people) rather than “person-first language” (e.g., people with Autism) (Sinclair, 2013).

## Appendix

APPENDIX 1 Day 1 interview questions for parents.

**Table:**

**What we say/do**	**Why we say/do it**
First of all, thank you for agreeing to take part in this study. We hope you’ll find it interesting. We’re going to be asking you some questions, but remember, there are no right or wrong answers. We just want to hear what you think. We’ll be glad to answer any questions you have, now or later.	Introduce ourselves.
You’ll notice we are using a camera to video-tape this session. We use it because we do not want to miss anything you say. If at anytime you wish not to be recorded, please let us know and we’ll turn off the camera.	Make participants comfortable; let them understand their rights.
How old is your child? What are some activities your child enjoys doing by themselves? What are some activities you enjoy doing with your child? What are some activities your child enjoys doing with other family members?	Better understand the relationship of child and family.
What would you tell a teacher or new person meeting your child about how they can avoid upsetting them? What are your child’s triggers? What does your child do when they are upset?	Identify signs and cues of distress in case they come up in the course of the study.
How frequently does your child produce repetitive behaviors? What types of repetitive behaviors does your child produce? How long does your child typically engage in repetitive behaviors for? When does your child stop stimming?	Better understand the nature of the child’s repetitive behaviors.
Does your child listen to music? What genres of music does your child listen to? What are some of your child’s current favorite songs/pieces/sounds? Does your child play a musical instrument?	Better understand the child’s relationship with sound.
What textures does your child like and dislike touching? Does your child like or dislike touching others (e.g., holding hands, hugging)? How can we tell if your child is uncomfortable interacting in this environment?	Better understand the child’s touch preferences.
